# Using Multiple Types of Studies in Systematic Reviews of Health Care Interventions – A Systematic Review

**DOI:** 10.1371/journal.pone.0085035

**Published:** 2013-12-26

**Authors:** Frank Peinemann, Doreen Allen Tushabe, Jos Kleijnen

**Affiliations:** 1 University of Maastricht, School for Public Health and Primary Care, Maastricht, The Netherlands; 2 Children's Hospital, University of Cologne, Cologne, Germany; 3 University of Birmingham, Department of Public Health, Epidemiology & Biostatistics, Birmingham, United Kingdom; 4 Kleijnen Systematic Reviews Ltd, York, United Kingdom; University Hospital Basel, Switzerland

## Abstract

**Background:**

A systematic review may evaluate different aspects of a health care intervention. To accommodate the evaluation of various research questions, the inclusion of more than one study design may be necessary. One aim of this study is to find and describe articles on methodological issues concerning the incorporation of multiple types of study designs in systematic reviews on health care interventions. Another aim is to evaluate methods studies that have assessed whether reported effects differ by study types.

**Methods and Findings:**

We searched PubMed, the Cochrane Database of Systematic Reviews, and the Cochrane Methodology Register on 31 March 2012 and identified 42 articles that reported on the integration of single or multiple study designs in systematic reviews. We summarized the contents of the articles qualitatively and assessed theoretical and empirical evidence. We found that many examples of reviews incorporating multiple types of studies exist and that every study design can serve a specific purpose. The clinical questions of a systematic review determine the types of design that are necessary or sufficient to provide the best possible answers. In a second independent search, we identified 49 studies, 31 systematic reviews and 18 trials that compared the effect sizes between randomized and nonrandomized controlled trials, which were statistically different in 35%, and not different in 53%. Twelve percent of studies reported both, different and non-different effect sizes.

**Conclusions:**

Different study designs addressing the same question yielded varying results, with differences in about half of all examples. The risk of presenting uncertain results without knowing for sure the direction and magnitude of the effect holds true for both nonrandomized and randomized controlled trials. The integration of multiple study designs in systematic reviews is required if patients should be informed on the many facets of patient relevant issues of health care interventions.

## Introduction

A systematic review may evaluate different aspects of a health care intervention such efficacy, effectiveness, and adverse events [1]. To accommodate the evaluation of various research questions such as efficacy or effectiveness and outcomes such as survival or severe adverse events, the inclusion of more than one study design appears to be necessary. If multiple study designs are included in a systematic review they should be well selected and customized to answer to the questions of interest. Efficacy addresses the question whether the intervention of interest can work in the ideal study setting (randomized controlled trial) and typically provides a conclusion for an average patient only [2]. In some situations RCTs are not feasible due to ethical concerns or due to strong patients' preferences and the results may not be applicable to everyday practice [3]. Some nonrandomized studies are designed to evaluate effectiveness and may show that interventions will work under every day circumstances, for example in a general practice [4]. Effectiveness typically provides a conclusion for a subgroup of patients that can be applied to individual patients. Adverse events can be crucial for approval, the restriction of application to particular indications, or the discontinuation of drugs or other interventions. The comprehensive detection of adverse events may need a long-term observation of a large number of participants and an experimental research design could become a costly and unsuccessful enterprise. It appears that many public commissioners provide predominantly funding for efficacy research [4].

A considerable proportion of researchers appears dichotomized to either require the randomized design for scientific evidence on health care interventions or to also accept designs without randomization as sufficient [5]. A 'hierarchy of evidence' was established that clearly downgrades designs other than randomized studies regardless of the type of outcome evaluated [6]. Some authors questioned this hierarchy [7,8]. Advantages and disadvantages of various designs have been reported repeatedly and some authors support the integration of multiple study designs with respect to the outcome of interest [5]. We did not find a report that systematically summarized methods papers about usefulness and complexity of integrating various designs in one systematic review. Therefore, we wanted to collect experiences, recommendations, and evidence based on clinical study data reported by others to infer whether one design is superior to others or not and whether alternative or more practical designs could complement or even replace a seemingly favorable design. One aim of this study is to find and describe articles on methodological issues concerning the incorporation of multiple types of study designs in systematic reviews on health care interventions. Another aim is to evaluate methods studies that have assessed whether reported effects differ by study types. Finally, we aimed to identify and summarize qualitative evidence sufficient enough to guide finding and integrating the right research design for answering various clinical questions within systematic reviews of health care interventions.

## Methods

While preparing this systematic review, we endorsed the PRISMA statement, adhered to its principles and conformed to its checklist (Table **S1**).

### Inclusion criteria

We included articles reporting on how to integrate different study designs in systematic reviews of health care interventions. We did not include articles merely describing advantages and disadvantages of various designs. We also included articles reporting different results of a particular outcome that depend on the type of design such as in a comparison of a randomized vs. a nonrandomized controlled design. Since we concentrated on the reporting of various study designs, we did not specify on the type of participants, interventions, comparisons, outcomes. 

### Search strategy

We searched PubMed, the Cochrane Database of Systematic Reviews, and the Cochrane Methodology Register on 31 March 2012. The search strategy is detailed in Table **1**. Terms and syntax used for the search in PubMed were also used for the Cochrane Libarary. The MeSH term "Randomized Controlled Trials as Topic"[MeSH] aims to specifically identify RCTs [9] while the MeSH term "Epidemiologic Studies"[Mesh] comprises nonrandomized study designs [10]. We combined terms of the controlled vocabulary MeSH with text words. We searched PubMed and the *Related citations* function in PubMed tool to find some pertinent articles that appeared to represent the topic of the present revew. We adopted candidate text words reported by those articles in the title or the abstract to build a search strategy for nonrandomized or observational studies [11-13]. 

**Table 1:** Search strategy.

**No**	**Term**
1	"Randomized Controlled Trials as Topic"[Mesh]
2	randomized controlled[tiab]
3	randomised controlled[tiab]
4	randomization[tiab]
5	randomisation[tiab]
6	random allocation[tiab]
7	"Epidemiologic studies"[Mesh] non random*[tiab]
8	nonrandom*[tiab]
9	non-random*[tiab]
10	observational[tiab]
11	quasi-experiment*[tiab]
12	quasi experiment*[tiab]
13	or/1-6
14	or/7-12
15	and/13-14

Searching PubMed, Cochrane database of systematic reviews, and the Cochrane database of methods studies on 31 March 2012.

Abbreviations and symbols. *: The asterisk represents truncation to find all terms that begin with a given text string; [Mesh]: Search field tag provided by PubMed for the search in Medical Subject Headings (MeSH) terms; [tiab]: Search field tag provided by PubMed for the search in the title and/or in the abstract

### Study selection

We imported the bibliographic data of the search results into an EndNote X4 database. Two reviewers assessed independently title and/or abstract whether randomized controlled trials and nonrandomized studies were addressed at the same time in any type of article. Disagreements were resolved by discussion. Full texts were ordered if we agreed on potentially relevant references and if disagreements could not be resolved. The full text papers were assessed to see whether the methodology of how to integrate specific study designs in systematic reviews was addressed. We also marked studies that compared the results of randomized controlled trials and nonrandomized studies on the same clinical topic to estimate possible effect size differences between the two design categories. 

### Data collection, analysis, and synthesis

We summarized the identified statements in a descriptive manner and did not quantitatively pool any data. We worked with 2 types of reviews, systematic reviews and other reviews. The systematic review category included Cochrane systematic reviews, other systematic reviews not issued by Cochrane, and health technology assessments. The other review category included non-systematic reviews, editorials, comments, and letters. We based the rationale to include non-systematic type papers on the following reflections. We wanted to build a comprehensive review of available methods papers. We wanted to acknowledge experience-based thoughts and reasonings and we wanted to include rationales and recommendations with respect to integrate various designs in systematic reviews that have been developed by others. We did not expect a large number of systematic reviews and we apprehended a limited scope of topics if we would have confined the data collection to systematic reviews only. Nevertheless, we stratified the results presentation by the two review types.

We identified 16 separately reported clinical fields and we used one additional category for articles that combined two or more clinical fields. The 17 categories were:

• Acupuncture: Intervention regarding acupuncture type of complementary and alternative medicine)
• Cardiology: Interventional procedures to reopen coronary arteries as opposed to surgical interventions
• Genetics: Genetic diseases and rare diseases
• HRT: Hormone replacement therapy for women
• Mental: Intervention to treat a mental disease such as depression
• Nephrology: Intevention regarding renal disease
• Nutrition: Influence of food on health
• Orthopedics: Intervention regarding orthopedic disease
• Palliation: Intervention regarding palliative treatment
• Pediatrics: Intervention regarding children
• Pharma: Drugs to treat patients
• Pregnancy: Intervention regarding pregnant women
• Social: Complex social interventions
• Surgery: Surgical intervention regarding various diseases
• Tele: Intervention regarding telehealth issues
• Transplant: Autologous or allogeneic transplantation of organs
• Various: Two or more different clinical fields

We created 8 distinct categories for classifying the type of study design:

• RCT: Randomized controlled trial
• NRCT: Nonrandomized controlled trial: prospective comparative trial with allocation of patients by physician
• Cohort study: Prospective or retrospective observational study with a control group without allocation of patients by physician, start is intervention
• CCS: Case-control study: retrospective study, start is events
• Regist: Registry of data from patients with particular diseases or interventions
• Admin: Administrative databases such as data from health care providers
• Survey: Survey or audit as well as postmarketing analysis
• Cases: Single case or case series

We identified a considerable number of different methodological topics relevant for the integration of various study designs in systematic reviews. As some of the topics were similar, we assigned these topics to 15 methodological categories. All major issues such as validity, applicability, and confounding were addressed in the papers.

• Adherence: Patients may adhere to the prescription or may not take drugs or doses as wanted
• Adverse events: Patients may experience unwanted effects or events that are associated with the intervention
• Applicability: Results may not be generalized to patients that have different characteristics than the study population
• Case load: The number of patients with a particular disease or intervention admitted to a hospital or treated by a physician
• Confounding: A known or unknown factor that is associated with the intervention and influences the outcome
• Exclusions: Certain patients are excluded from the recruitment such as elderly, pregnant women, children, patients with comorbidities
• Heterogeneity: Patients within one treatment group differ in baseline characteristics such as severity of disease
• Long term: Follow up more than 12 months after the intervention
• Participation: Eligible individuals who did not participate in trials
• Pathophysiol: Pathophysiological issues such as bacterial cause or various genetic constitution
• Preferences: Patients and physicians may have preferences about what treatment is best
• Rare disease: Rare diseases may not be represented in clinical trials and rare adverse events may not be detected by small studies
• Specialisation: The level of education and experience of a physician may influence the outcome
• Survival: Proportion of patients that sustain a specific wanted status after a certain time period
• Validity: To measure what should be measured; minimizing uncertainty and systematic error; dealing with selection bias

## Results

### Search results

**Figure 1:**
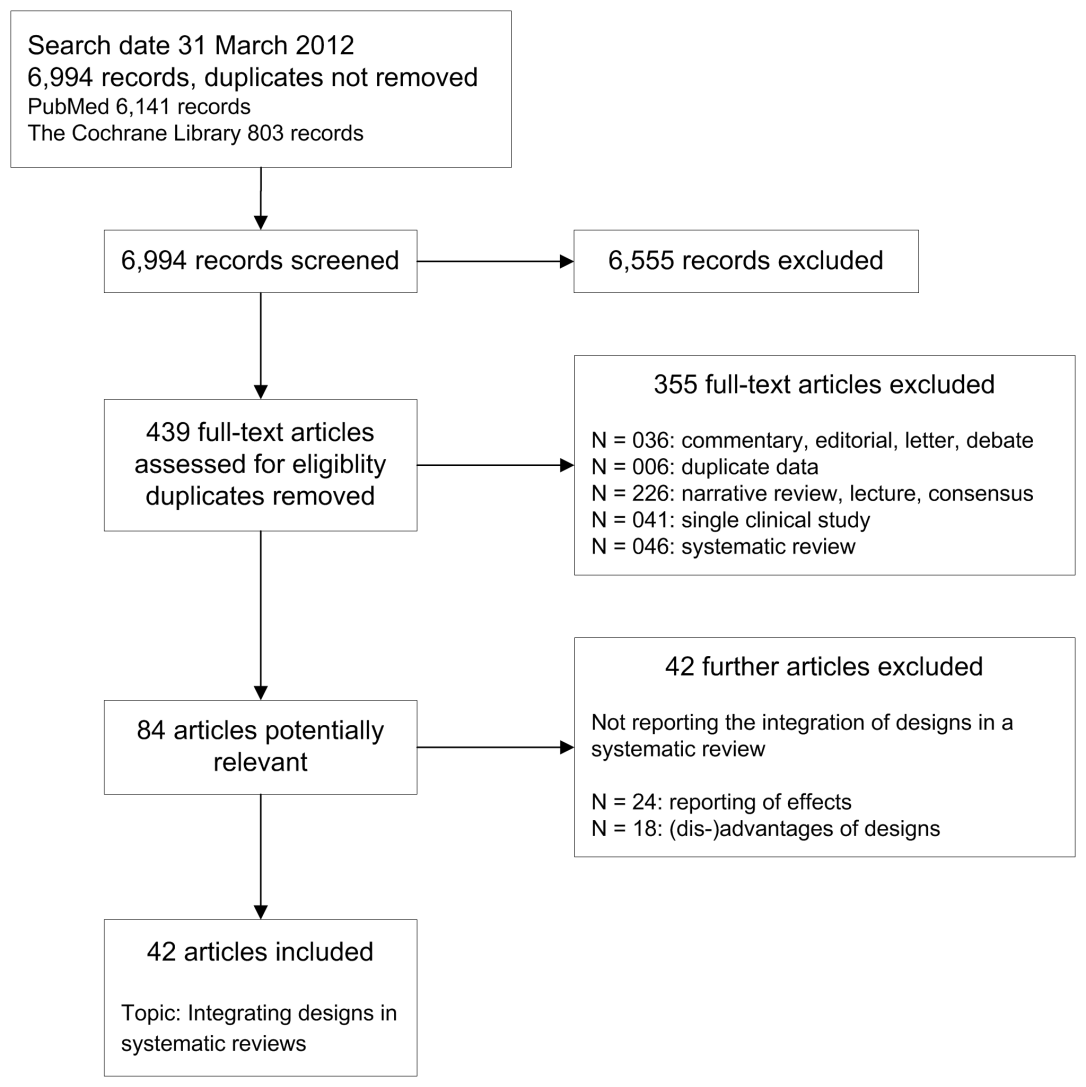
Literature retrieval and study selection.

We included 42 articles that report about the integration of study designs in systematic reviews (Figure **1**) [5,7,8,14-52]. In the first step of the study selection process, we retrieved 6994 records from electronic databases including 6141 citations from PubMed and 803 citations from the Cochrane Library. The Cochrane Library citations were made of 188 systematic reviews and 526 methods studies. After excluding 6555 records not relevant to the topic of interest or duplicates, we assessed the fulltexts of 439 different articles. After a first screening process, we excluded 355 articles and after a repeated screening of the remaining potentially relevant fulltexts, we excluded another 42 articles. The reasons for exclusion are shown in Figure **1**. 

### Characteristics of included articles

The characteristics of included articles are shown in Table **2** and Table **3**. We identified 8 systematic reviews [14,17-20,33,34,38] and 34 non-systematic reviews including editorials, comments, or letters. The articles containing concepts relevant to our research question were published between 1995 and 2012. Most of the articles were published between 2005 and 2012: 73% (31 of 42) of all reviews, 62% (5 of 8) systematic reviews and 76% (26 of 34) non-systematic reviews (Table **2**). The systematic reviews covered 4 of 16 distinct clinical field categories with 5 of 8 reviews reporting on surgery and with 1 review reporting on acupuncture, cardiology, and various clinical fields, respectively (Table **2**). The non-systematic reviews covered 15 of 16 categories with 12 reporting on various topics, 4 reporting on surgery, no report on acupuncture, and 1 to 2 reporting on each of the rest of clinical entities.

**Table 2:** Characteristics of included articles.

**Author**	**Year**		**Ref**	**Field**	**Type of design**							
					**RCT**	**NRCT**	**Cohort**	**CCS**	**Regist**	**Admin**	**Survey**	**Cases**
***Systematic review***												
Archampong	2012		[14]	Surgery		1	1	1	1		1	
Britton	1998		[17]	Surgery	1	1						
Chambers	2009		[18]	Cardiology	1	1						1
Chambers	2010		[18]	Surgery	1				1			
Chou	2010		[20]	Surgery	1	1	1	1	1	1	1	1
Lewsey	2000		[33]	Surgery	1					1		
Linde	2002		[34]	Acupuncture	1	1	1	1			1	1
Norris	2005		[38]	Various		1	1	1				1
***Non-systematic review***												
Atkins	2007		[15]	Surgery	1	1	1	1	1	1	1	
Black	1996		[16]	Various			1	1				
Chumbler	2008		[21]	Tele	1	1	1					1
Concato (Comp)	2010		[7]	Surgery	1	1	1			1		
Concato (Observ)	2010		[22]	Various	1	1	1	1				
Essock	2003		[23]	Mental	1	1						
Fletcher	2002		[24]	Various	1	1	1	1				
Fletcher	2009		[25]	HRT	1	1	1					
Gale	2009		[26]	Transplant	1				1			
Grzeskowiak	2012		[27]	Pregnancy			1	1	1	1		1
Hadley	2009		[61]	Palliation	1		1		1		1	
Hartling	2005		[29]	Surgery		1	1	1				1
Hodgson	2007		[30]	Mental	1		1					
Hoppe	2009		[8]	Orthopedics	1		1	1				1
Horn	2010		[31]	Various	1	1						
Kovesdy	2012		[32]	Nephrology	1		1					
McCarthy	2008		[35]	Surgery	1	1	1					1
Mercer	2007		[36]	Various	1	1						1
Mitchell	1995		[37]	Pediatrics	1							
Norris	2011		[39]	Various		1	1	1	1			1
Ogilvie	2005		[40]	Social	1	1	1	1				1
Olivier	2006		[41]	Pharma	1	1	1	1	1	1		1
Reeves	2005		[42]	Nutrition	1		1	1				
Rosendaal	2001		[43]	Cardiology	1			1				
Sharma	2012		[44]	Social	1		1					
Shrier	2007		[45]	Various	1		1					
Silverman	2009		[46]	Pharma	1		1	1		1		
Vandenbroucke	1998		[47]	Various	1			1				
Vandenbroucke	2004		[48]	Various	1			1				
Vandenbroucke	2008		[5]	Various	1			1				
Vandenbroucke	2009		[49]	HRT	1		1					
Vandenbroucke	2011		[50]	Various	1	1						
Wilcken	2001		[51]	Genetics	1		1		1			
Zlowodzki	2006		[52]	Various	1	1	1	1				1
	**Frequency**				**36**	**22**	**28**	**21**	**10**	**7**	**5**	**14**

Type of review. Systematic review (first 8 papers): Cochrane Systematic Review (Archampong 2012), Health Technology Assessment of National Health Service in UK (Britton 1998), other systematic reviews not issued by Cochrane or HTA (Chambers 2009, Chambers 2010, Chou 2010, Lewsey 2000, Linde 2002, Norris 2005). Non-systematic review (rest of 34 papers): narrative review or editorial or comment or letter.

Field. Acupuncture: Intervention regarding acupuncture type of complementary and alternative medicine; Cardiology: Interventional procedures to reopen coronary arteries as opposed to surgical interventions; Genetics: Genetic diseases and rare diseases; HRT: Hormone replacement therapy for women; Mental: Intervention to treat a mental disease such as depression; Nephrology: Intevention regarding renal disease; Nutrition: Influence of food on health; Orthopedics: Intervention regarding orthopedic disease; Palliation: Intervention regarding palliative treatment; Pediatrics: Intervention regarding children; Pharma: Drugs to treat patients; Pregnancy: Intervention regarding pregnant women; Social: Complex social interventions; Surgery: Surgical intervention regarding various diseases; Tele: Intervention regarding telehealth issues; Transplant: Autologous or allogeneic transplantation of organs; Various: Two or more different clinical fields

Type of design. RCT: Randomized controlled trial; NRCT: Nonrandomized controlled trial: prospective comparative trial with allocation of patients by physician; Cohor: Prospective or retrospective observational study without allocation of patients by physician, start is intervention; CCS: Case-control study: retrospective study, start is events; Regist: Registry of data from patients with particular diseases or interventions; Admin: Administrative databases such as data from health care providers; Survey: Survey or audit or postmarketing analysis; Cases: Single case or case series

Other abbreviations. Ref: reference

**Table 3:** Outcomes of included articles.

**Authors**	**Year**		**Ref**	**Outcomes addressed**														
				**Adherence**	**Adverse events**	**Applicability**	**Case-load**	**Confounding**	**Exclusions**	**Heterogeneity**	**Long term**	**Participation**	**Patho-physiol**	**Preferences**	**Rare disease**	**Special-isation**	**Survival**	**Validity**
***Systematic review***																		
Archampong	2012		[14]				1									1	1	
Britton	1998		[17]			1		1	1	1	1	1		1				
Chambers	2009		[18]		1	1					1							
Chambers	2010		[18]		1												1	
Chou	2010		[20]	1	1	1				1	1							1
Lewsey	2000		[33]			1												1
Linde	2002		[34]		1	1					1							
Norris	2005		[38]			1		1										1
***Non-systematic review***																		
Atkins	2007		[15]		1	1				1								
Black	1996		[16]	1	1	1					1	1		1	1			1
Chumbler	2008		[21]															1
Concato (Comp)	2010		[7]			1												1
Concato (Observ)	2010		[22]					1										1
Essock	2003		[23]			1		1			1			1				1
Fletcher	2002		[24]			1		1										1
Fletcher	2009		[25]					1										1
Gale	2009		[26]			1		1				1						1
Grzeskowiak	2012		[27]		1			1										1
Hadley	2009		[61]		1					1								
Hartling	2005		[29]		1	1					1							
Hodgson	2007		[30]		1	1			1		1	1						1
Hoppe	2009		[8]			1		1			1	1		1				1
Horn	2010		[31]			1												1
Kovesdy	2012		[32]	1		1		1		1	1							1
McCarthy	2008		[35]					1										1
Mercer	2007		[36]			1												1
Mitchell	1995		[37]		1			1										
Norris	2011		[39]			1		1		1	1			1				1
Ogilvie	2005		[40]			1			1									1
Olivier	2006		[41]		1						1							
Reeves	2005		[42]					1										1
Rosendaal	2001		[43]					1										1
Sharma	2012		[44]										1					
Shrier	2007		[45]					1										1
Silverman	2009		[46]	1		1					1							1
Vandenbroucke	1998		[47]		1			1					1					1
Vandenbroucke	2004		[48]		1			1										1
Vandenbroucke	2008		[5]		1			1			1							1
Vandenbroucke	2009		[49]		1			1	1									1
Vandenbroucke	2011		[50]		1													1
Wilcken	2001		[51]		1						1				1			
Zlowodzki	2006		[52]					1										1
	**Frequency**			**4**	**18**	**21**	**1**	**21**	**4**	**6**	**15**	**5**	**2**	**5**	**2**	**1**	**2**	**30**

Outcomes addressed. Adherence: Patients may adhere to the prescription or may not take drugs or doses as wanted; Adverse events: Patients may experience unwanted effects or events that are associated with the intervention; Applicability: Results may not be generalized to patients that have different characteristics than the study population; Case load: the number of patients with a particular disease or intervention admitted to a hospital or treated by a physician; Confounding: A known or unknown factor that is associated with the intervention and influences the outcome; Exclusions: Certain patients are excluded from the recruitment such as elderly, pregnant women, children, patients with comorbidities; Heterogeneity: Patients within one treatment group differ in baseline characteristics such as severity of disease; Long term: follow up more than 12 months after the intervention; Participation: Eligible individuals who did not participate in trials; Pathophysiol: Pathophysiological issues such as bacterial cause or various genetic constitution; Preferences: Patients and physicians may have preferences about what treatment is best; Rare disease: Rare diseases may not be represented in clinical trials and rare adverse events may not be detected by small studies; Specialisation: the level of education and experience of a physician may influence the outcome: Survival: Proportion of patients that sustain a specific wanted status after a certain time period; Validity: to measure what should be measured; minimizing uncertainty and systematic error, dealing with selection bias Other abbreviations. Ref: reference

Of the 15 methodological topics relevant for the integration of various study designs in systematic reviews, 5 topics were frequently reported by more than 10 articles (Table **3**). The rest were addressed by 1 article or up to 6 articles. Validity was reported by 30 reviews (systematic 3, non-systematic 27), applicability by 21 reviews (systematic 6, non-systematic 15), confounding by 21 reviews (systematic 2, non-systematic 19), adverse events by 18 reviews (systematic 4, non-systematic 14), and long-term follow up by 15 reviews (systematic 4, non-systematic 11). Systematic reviews reported 13 categories leaving pathogenesis and rare diseases out. Non-systematic reviews reported 12 categories and did not refer to case load, specialisation, and survival.

### Key messages

We qualitatively summarized the key messages of the 42 included methods studies based on the extraction of major statements (Table **S2**). We identified a clear tendency in the message that nonrandomized studies should be conducted and integrated in systematic reviews to complement available RCTs or replace lacking RCTs in 85% (36 of 42) of all reviews. We judged the difference between systematic reviews 75% (6 of 8) and non-systematic reviews 88% (30 of 34) as not considerable. Thus the majority of identified reviews supported the view that nonrandomized studies are important and should be an integral part of assessing health care interventions. Only a minority of reviews regarded RCTs as the sole means of finding reliable answers to clinical research questions. Most papers acknowledged the advantages and the disadvantages of RCTs and nonrandomized studies with regard to specific methodologic topics or specific clinical outcomes. Some papers addressed the problem that RCTs are not possible for assessing certain questions and that case reports may have a considerable impact on safety issues.

### Comparison of randomized vs. nonrandomized controlled design

We identified 49 studies, 18 trials and 31systematic reviews that compared the effect measures found in randomized controlled trials with those in nonrandomized controlled trials (Table **4**). Of these 49 studies, 39 reported about the same or similar intervention in both study designs and 10 studies that included different interventions in the analyses. In 35% (17 of 49) studies, there was a different direction or a statistically significant difference of the magnitude of effect between randomized and nonrandomized controlled trials. In 53% (26 of 49) studies, the effect did not differ considerably between those two designs. In 12% (6 of 49) studies, both results, a difference as well as no difference were reported.

**Table 4:** Reviews and studies comparing randomized vs. nonrandomized controlled results.

**First author**	**Year**	**Ref**	**Intervention**	**Difference R vs. N**		
				**Yes**	**Various**	**No**
Abraham	2010	[62]	same			no
Algra	2012	[63]	same			no
Antman	1985	[64]	same	yes		
Aslani	2010	[65]	same			no
Benis	2002	[66]	same	yes		
Benson	2000	[67]	same			no
Bhandari	2004	[68]	same	yes		
Britton	1998	[17]	same			no
Carroll	1996	[69]	same	yes		
CASS	1984	[70]	same			no
Cheng	2012	[71]	same			no
Choi	2012	[72]	same			no
Clagett	1984	[73]	same			no
Colditz	1989	[74]	various	yes		
Conaty	2004	[75]	same			no
Concato	2000	[76]	same			no
Deeks	2003	[77]	various		various	
Edwards	2012	[78]	various			no
Flossman	2007	[79]	same			no
Franklin	2000	[80]	same			no
Furlan (Exam)	2008	[81]	same		various	
Furlan (Meth)	2008	[82]	same	yes		
Golder	2011	[83]	various			no
Gross	2005	[84]	same			no
Guyatt	2000	[85]	same	yes		
Hannan	2008	[86]	various		various	
Hlatky	1988	[87]	same			no
Ioannidis	2001	[58]	various	yes		
Kunz	2007	[54]	same	yes		
Kunz	1998	[53]	same	yes		
Kuss	2011	[88]	same			no
Lawlor	2004	[89]	same	yes		
Linde	2002	[34]	same			no
MacLehose	2000	[11]	various			no
Mueller	2010	[90]	same	yes		
Naudet	2011	[91]	same	yes		
Odgaard-Jensen	2011	[55]	various		various	
Papanikolaou	2006	[92]	various		various	
Phillips	1999	[93]	same			no
RMIT	1994	[94]	same			no
Rovers	2001	[95]	same			no
Schmoor	2008	[96]	same			no
Shea	2010	[97]	same	yes		
Shikata	2006	[98]	various		various	
Tzoulaki	2011	[99]	same	yes		
Vis	2008	[100]	same	yes		
Vist	2008	[101]	same			no
Wilkes	2010	[102]	same			no
Wolfe	2004	[103]	same	yes		
**Frequency**				**17**	**6**	**26**

Abbreviations: CASS: Coronary artery surgery study; Difference R vs N: difference between randomized vs. nonrandomized results; Ref: reference; RMIT: Recurrent Miscarriage Immunotherapy Trialists

## Discussion

We identified and summarized qualitative evidence sufficient enough to guide finding and integrating the right research design for answering various clinical questions within the conduct of systematic reviews of health care interventions.

It is obvious that intended effects of interventions such as the physician-reported outcomes of prevention of death and healing or improving of disease in ideal settings with financially affordable follow up and with ample number of available participants are best investigated in well planned RCTs. There is no equal or better alternative study design. The results may or may not be applicable to the general population. Many people with particular characteristics such as younger or older age, gender, pregnancy, or comorbidity may have been excluded and may have experienced opposing effects or an unfavorable and unwanted balance of benefit and harm. Pediatricians may seek information on drugs from observational studies if data on the treatment of children from RCTs are not available. Unintended, severe adverse events require long-term observation including postmarketing analysis, administrative databases, and case reports to identify harmful drugs that have to be withdrawn from the market. The types of different study design that need to be included in a systematic review depend on the nature of the clinical questions that the review addresses.

Oxman and collaborators assessed the effects of randomisation and concealment of allocation on the results of healthcare studies and reported their results in three papers within the time period from 1998 to 2011 [53-55]. The authors concluded that "the results of randomised and non-randomised studies – sometimes – differed". In many cases the results did not differ. The authors argued "that it is not generally possible to predict the magnitude, or even the direction, of possible selection biases and consequent distortions of treatment effects from studies with non-random allocation or controlled trials with inadequate or unclear allocation concealment". We believe that trials with random allocation and adequate allocation concealment may show contradictory results. We also believe that it is not possible to foresee the magnitude or the direction of bias in those adequately randomized trials with absolute certainty [56]. Nevertheless, the authors stated that "randomized controlled trials are a safeguard against biased estimates of treatment effects". Various design prerequisites and adjustment procedures in nonrandomized controlled trials can minimize bias and confounding, however, it is not kown for certain in a particular trial whether the results reflect the reality or whether they are distorted. The same principle holds true for trials with adequate randomization and concealment of allocation. Even if the risk of a false estimate determined in a series of trials would be lower than in trials with inadequate randomization and concealment of allocation the fact is that the result of the primary outcome measure in a single specific trial cannot be regarded as an absolute and certain proof regardless of the p-values or confidence intervals. Ioannidis 2005 concluded that, quote: "Controversies are most common with highly cited nonrandomized studies, but even the most highly cited randomized trials may be challenged and refuted over time, especially small ones" [57]. The authors found that 5 of 6 highly cited nonrandomized studies had been contradicted or had found stronger effects versus 9 of 39 randomized controlled trials (P = 0.008). Our assessment adds to the existing work done by Oxman group and the Ioannidis group that the effect did not differ considerably between the randomized and the nonrandomized designs in more than half of the studies. The general postulate or dogma of the RCT as a safeguard against biased estimates of treatment effects may create deceptive promises and may give researchers a false sense of security. We infer from our findings just the same as Shrier 2007 has expressed before, quote: "(...)that excluding observational studies in systematic reviews a priori is inappropriate and internally inconsistent with an evidence-based approach" [45].

According to the Cochrane handbook, the Cochrane Collaboration focuses particularly on systematic reviews of RCTs and considers inclusion of nonrandomized studies mainly if RCTs are lacking. We see a vast number of clinical research questions that are not investigated by RCTs. There may be many reasons, for example, patients' and physicians' preferences that prevent the accumulation of true randomized study data. Our results suggest that the Cochrane Collaboration might be advised to consider more reasons for including nonrandomized studies on the condition of a rigorous risk of bias assessment and confinement to specific interventions and outcomes.

In general, a high risk of bias is inherent in all nonrandomized studies. Certain study characteristics such as prospective design, concurrent control group, adjustment of results with respect to different baseline values, and confounder control can limit additional bias. For example, Ioannidis 2001 [58] reported that discrepancies between RCT and nonrandomized studies were less common when only nonrandomized studies with a prospective design were considered. The Cochrane Collaboration offers a guide for inclusion of nonrandomized studies [59] and it has developed a tool for assessing the risk of bias in both RCT and controlled nonrandomized studies[60]. 

## Conclusions

Different study designs addressing the same question yielded varying results, with differences in about half of all examples. The risk of presenting uncertain results without knowing for sure the direction and magnitude of the effect holds true for both nonrandomized and randomized controlled trials, though, the risk of bias and confounding is probably higher in the nonrandomized ones. The integration of multiple study designs in systematic reviews is required if patients should be informed on the many facets of patient relevant issues of health care interventions.

## Supporting Information

Table S1 **PRISMA Checklist.**
(DOC)

Table S2 **Qualitative summary of key messages.** Type of review. Systematic review (first 8 papers): Cochrane Systematic Review (Archampong 2012), Health Technology Assessment of National Health Service in UK (Britton 1998), other systematic reviews not issued by Cochrane or HTA (Chambers 2009, Chambers 2010, Chou 2010, Lewsey 2000, Linde 2002, Norris 2005). Non-systematic review (rest of 34 papers): narrative review or editorial or comment or letter.
Message. We extracted messages with respect to the question whether nonrandomized studies should be conducted or integrated in systematic reviews to complement available RCTs or replace lacking RCTs. We did not extract data on differences between those two study design on size or direction of effect.
NRS also: We perceived a tendency in the message that nonrandomized studies should also be considered in addition to RCTs in general or to answer specific research questions.
RCT only: We perceived a tendency in the message that RCTs are sufficient to answer research questions in clinical trials and in systematic reviews and that nonrandomized studies cannot complement or replace them.
Field. Acupuncture: Intervention regarding acupuncture type of complementary and alternative medicine; Cardiology: Interventional procedures to reopen coronary arteries as opposed to surgical interventions; Genetics: Genetic diseases and rare diseases; HRT: Hormone replacement therapy for women; Mental: Intervention to treat a mental disease such as depression; Nephrology: Intevention regarding renal disease; Nutrition: Influence of food on health; Orthopedics: Intervention regarding orthopedic disease; Palliation: Intervention regarding palliative treatment; Pediatrics: Intervention regarding children; Pharma: Drugs to treat patients; Pregnancy: Intervention regarding pregnant women; Social: Complex social interventions; Surgery: Surgical intervention regarding various diseases; Tele: Intervention regarding telehealth issues; Transplant: Autologous or allogeneic transplantation of organs; Various: Two or more different clinical fields.
Other abbreviations. Ref: reference.
(DOCX)
