# Supplementary material for: Using Multiple Types of Studies in Systematic Reviews of Health Care Interventions – A Systematic Review
Source: PLoS One. 2013 Dec 26;8(12):e85035. doi: 10.1371/journal.pone.0085035 (PMC3887134; doi:10.1371/journal.pone.0085035)
Supplement: Table S2 — Qualitative summary of key messages. Type of review. Systematic review (first 8 papers): Cochrane Systematic Review (Archampong 2012), Health Technology Assessment of National Health Service in UK (Britton 1998), other systematic reviews not issued by Cochrane or HTA (Chambers 2009, Chambers 2010, Chou 2010, Lewsey 2000, Linde 2002, Norris 2005). Non-systematic review (rest of 34 papers): narrative review or editorial or comment or letter. Message. We extracted messages with respect to the question whether nonrandomized studies should be conducted or integrated in systematic reviews to complement available RCTs or replace lacking RCTs. We did not extract data on differences between those two study design on size or direction of effect. NRS also: We perceived a tendency in the message that nonrandomized studies should also be considered in addition to RCTs in general or to answer specific research questions. RCT only: We perceived a tendency in the message that RCTs are sufficient to answer research questions in clinical trials and in systematic reviews and that nonrandomized studies cannot complement or replace them. Field. Acupuncture: Intervention regarding acupuncture type of complementary and alternative medicine; Cardiology: Interventional procedures to reopen coronary arteries as opposed to surgical interventions; Genetics: Genetic diseases and rare diseases; HRT: Hormone replacement therapy for women; Mental: Intervention to treat a mental disease such as depression; Nephrology: Intevention regarding renal disease; Nutrition: Influence of food on health; Orthopedics: Intervention regarding orthopedic disease; Palliation: Intervention regarding palliative treatment; Pediatrics: Intervention regarding children; Pharma: Drugs to treat patients; Pregnancy: Intervention regarding pregnant women; Social: Complex social interventions; Surgery: Surgical intervention regarding various diseases; Tele: Intervention regarding telehealth issues; Transplant: Autologous or [file pone.0085035.s002.docx]

Table S2. Qualitative summary of key messages

| **Author** | **Year** | **Ref** | **Field** | **Message** | **NRS also** | **RCT only** |
| --- | --- | --- | --- | --- | --- | --- |
| ***Systematic review*** |  |  |  |  |  |  |
| Archampong | 2012 | [[14](#_ENREF_14)] | Surgery | In the absence of RCTs, overall five year survival was significantly improved for patients with colorectal cancer treated in high-volume hospitals, by high-volume surgeons and colorectal specialists in observational studies. | Yes |  |
| Britton | 1998 | [[17](#_ENREF_17)] | Surgery | Results of RCTs and NRS do not inevitably differ; subjects included in each type of study should be comparable. A well-designed non-randomised study is preferable to a small, poorly designed and exclusive RCT. Problems in RCTs may be non-participation of patients and physicians. | Yes |  |
| Chambers | 2009 | [[18](#_ENREF_18)] | Cardiology | Inclusion of case series can increase the evidence base and strengthen the credibility of a review | Yes |  |
| Chambers | 2010 | [[18](#_ENREF_18)] | Surgery | Three registries did not support the hypothesis that the findings of six RCTs are not representative of clinical practice. |  | Yes |
| Chou | 2010 | [[20](#_ENREF_20)] | Surgery | Appropriate synthesis of harms data must also consider issues related to evaluation of rare or uncommon events, assessments of equivalence or noninferiority, and use of indirect comparisons. | Yes |  |
| Lewsey | 2000 | [[33](#_ENREF_33)] | Surgery | RCT is not possible to study early versus late surgery for subarachnoid haemorrhage. RCT is not ethical to study the influence of co-morbidities on long-term effects of transurethral prostatectomy. A meta-analysis of RCTs that studies coronary artery bypass grafting could be augmented. Bayesian analyses may provide a link between RCTs that are unbiased by design and routine data that are biased but reflect real populations. | Yes |  |
| Linde | 2002 | [[34](#_ENREF_34)] | Acupuncture | 35 NRS (5 cohort studies, 10 prospective uncontrolled studies, 10 case series, and 10 cross-sectional surveys) confirmed the results from RCTs that the treatment is likely to be effective but provided little relevant additional information on long-term effects and adverse effects |  | Yes |
| Norris | 2005 | [[38](#_ENREF_38)] | Various | In surgery, public health, and the organization of health care delivery, most evidence rests on NRS. The inclusion of different study designs varies with the nature of the clinical question. Systematic reviews of important treatment questions will frequently need to consider non-randomized studies. | Yes |  |
| ***Non-systematic review*** |  |  |  |  |  |  |
| Atkins | 2007 | [[15](#_ENREF_15)] | Surgery | Research using cohort and case-control designs, disease and intervention registries, and outcomes studies based on administrative data can all shed light on who is most likely to benefit from the treatment, and what the important tradeoffs are. Other research designs than RCTs can provide important evidence to strengthen our understanding of how to apply research findings in practice. | Yes |  |
| Black | 1996 | [[16](#_ENREF_16)] | Various | Observational methods are needed both to evaluate the parts randomised trials cannot reach and to interpret correctly the results obtained from these trials. RCTs can be UNNECESSARY: 1. effect of an intervention is dramatic. RCTs can be INAPPROPRIATE: 1. rarely large enough to measure accurately infrequent adverse outcomes; 2. evaluting interventions to prevent rare events; 3. outcomes of interest are far in the future; 4. act of random allocation may reduce the effectiveness. RCTs can be IMPOSSIBLE: 1. refusal of clinicians to participate; 2. ethical objections; 3. political obstacles; 4. legal obstacles; 5. some interventions cannot be allocated on a random basis; 6. contamination; 7. scale of the task. RCTs can be INADEQUATE (applicability): 1. health care professionals unrepresentative; 2. patients atypical; 3. treatment atypical. For too long a false conflict has been created between those who advocate randomised trials in all situations and those who believe observational data provide sufficient evidence. | Yes |  |
| Chumbler | 2008 | [[21](#_ENREF_21)] | Tele | In the absence of a pure RCT in a telehealth study, there are several quasi-experimental design techniques such as retrospective matched cohort study design that can be implemented as viable alternatives. | Yes |  |
| Concato (Comp) | 2010 | [[7](#_ENREF_7)] | Surgery | Rather than adhering to a hierarchy of research design, comparative effectiveness research should be conducted and evaluated using sound clinical, epidemiological, and statistical judgment. | Yes |  |
| Concato (Observ) | 2010 | [[22](#_ENREF_22)] | Various | Case series or historical controlled trials would not be considered well designed. RCTs are almost always superior to observational studies. Contradictions among RCTs themselves are well described. Specific aspects of any particular study can be more important than the category of study design as randomized versus observational. | Yes |  |
| Essock | 2003 | [[23](#_ENREF_23)] | Mental | RCTs are expensive in time and money and must compare simple differences in treatments. Findings have a high internal validity but may not address the needs of the field, particularly where treatment is complex and rapidly evolving. | Yes |  |
| Fletcher | 2002 | [[24](#_ENREF_24)] | Various | Observational studies are often, but not always, an adequate substitute. When trials disagree, resolution may be found not by conducting more trials but from critical appraisal of individual trials. | Yes |  |
| Fletcher | 2009 | [[25](#_ENREF_25)] | HRT | Complex diseases require complex evidence with diverse types of studies from diverse populations. Portfolio-type reviews are more demanding than the hierarchy of evidence approach because they require careful appraisal and synthesis of results from a wide range of study designs. | Yes |  |
| Gale | 2009 | [[26](#_ENREF_26)] | Transplant | Observational databases and structured quantification of expert opinion have an important role in answering therapy questions in blood cells and bone marrow transplantation and that results of these studies should not be discounted simply because they are not RCTs. | Yes |  |
| Grzeskowiak | 2012 | [[27](#_ENREF_27)] | Pregnancy | In the absence of RCTs, the best means of evaluating medication safety during pregnancy is provided by NRS such as case reports, cohort studies (prospective pregnancy exposure registries and administrative database studies), case-control studies, or nested case-control studies. | Yes |  |
| Hadley | 2009 | [[61](#_ENREF_61)] | Palliation | 91 studies with 200 and more participants were identified. Of those, 1 study was an RCT and 90 were observational studies lacking good quality. | Yes |  |
| Hartling | 2005 | [[29](#_ENREF_29)] | Surgery | Use RCTs to examine treatment effect, when available. Include nonrandomized studies with a control group only when RCTs are sparse or unavailable; randomized evidence is especially lacking for devices and surgical procedures. Use nonrandomized studies to complement RCT evidence for long-term events and safety outcomes. RCTs tend to enroll younger and healthier patients; the in-hospital mortality rates after carotid endarterectomy are almost 10-fold higher in the "real-world" setting than in the trials included in the systematic review. | Yes |  |
| Hodgson | 2007 | [[30](#_ENREF_30)] | Mental | Outcomes of RCTs often lack clinical utility and usually do not address realworld effectiveness. Older patients, women, and patients with comorbid disorders are underrepresented in and adverse events may be reported inadequately in RCTs. If masking is not possible or ethical concerns preclude randomization then observational studies might represent the only method for studying certain aspects of treatment. No current research design provides comprehensive clinical information. | Yes |  |
| Hoppe | 2009 | [[8](#_ENREF_8)] | Orthopedics | While a randomized controlled trial is the most objective design, it may not provide all of the information a clinician might need. Questions concerning the etiology of a disease, its natural history, the identification of prognostic factors, and the possibility of adverse treatment effects can be addressed through carefully designed observational studies with good inclusion criteria for selecting patients. Blinding is rather difficult to achieve in surgery. It would be wrong to randomize patients to an unhealthy or healthy diet, to evaluate in an experimental design the effect of smoking on the healing of open tibial fractures and the effect of inhaled corticosteroids on the subsequent risk of fracture in children and adolescents. Long-term outcome and prognosis are particularly important in orthopaedics because implants such as hip and knee prostheses now last many years. RCTs only answer the first question (Can it work?) and not the rest of two questions (Will it work? Is it worth it?) posed by Cochrane. | Yes |  |
| Horn | 2010 | [[31](#_ENREF_31)] | Various | Studies with a practice-based evidence for clinical practice improvement (PBE-CPI) are weaker than RCTs on internal validity but stronger on external validity. | Yes |  |
| Kovesdy | 2012 | [[32](#_ENREF_32)] | Nephrology | Absolute certainty cannot ever be attained in medicine. That shall also be true for properly designed and conducted RCTs. The possible problems of RCTs include the nonadherence to an assigned intervention, the inability to follow up on enrolled participants, the creation of confounding when characteristics differ between two treatment groups, the pooling of many poor-quality studies does not render the aggregate results qualitatively more valid than those of the individual studies themselves, the heterogeneity among the individual studies included in the meta-analysis can make it difficult to unify their findings under a single umbrella. | Yes |  |
| McCarthy | 2008 | [[35](#_ENREF_35)] | Surgery | Cases can be used to generate hypotheses, communicate rare events, and provide descriptive reports that may be useful when defining or modifying existing techniques. RCTs provide the most rigorous way of determining whether a cause-effect relationship exists between treatment and outcome. Although many issues make surgical RCTs more challenging to perform, they rarely preclude their performance. As plastic surgeons, we can move away from the retrospective reporting of cases and nonrandomized studies and instead rely on prospective, randomized trials addressing important clinical issues. |  | Yes |
| Mercer | 2007 | [[36](#_ENREF_36)] | Various | RCT remains the gold standard for safeguarding internal validity. NRS can address external validity and is the second choice when randomization or experimental control or both are impossible or inadequate. The choice of study design is shaped by the specific research question among other issues. | Yes |  |
| Mitchell | 1995 | [[37](#_ENREF_37)] | Pediatrics | How to identify rare but serious reactions to pediatric ibuprofen? NRS are inadequate in the presence of confounding by indication, that is, when treated with a drug, patients differ in their underlying risk of adverse outcome from patients given alternative treatments independent of the effect of the drug. A rigorous and large RCT is needed to provide valid estimates of rare but serious adverse events. |  | Yes |
| Norris | 2011 | [[39](#_ENREF_39)] | Various | Because it is unusual to find sufficient evidence from RCTs to answer all key questions concerning benefit or the balance of benefits and harms, comparative effectiveness reviewers should routinely assess the appropriateness of inclusion of observational studies for questions of benefit. | Yes |  |
| Ogilvie | 2005 | [[40](#_ENREF_40)] | Social | Relying on RCTs would have seriously compromised the scope and value of our evidence synthesis. Filtering out studies for exclusion without examining them in detail may deprive both reviewers and users of important evidence and insights. A report of a systematic review is no different from any other scientific publication: it can give a misleading narrative of the research process. Hierarchy of study design as a means of selecting studies may reduce the value of evidence synthesis. | Yes |  |
| Olivier | 2006 | [[41](#_ENREF_41)] | Pharma | A total of 21 drugs were withdrawn for safety reasons between 1998 and 2004 in France. For 19 out of 21 drugs, scientific evidence leading to drug withdrawal came from spontaneous case reports (or case series). In only one case, a RCT supported the decision. A pharmaco-epidemiological population-based database could represent a helpful tool to generate, test and quantify safety hypothesis. | Yes |  |
| Reeves | 2005 | [[42](#_ENREF_42)] | Nutrition | Nutritional choices and uptake of health education about nutrition are very likely to be associated with potential confounding factors when exposure is compared in NRS. Pooled estimates of the effects of nutritional exposures and their confidence intervals are likely to be misleading. There are many reasons for caution such as selection bias, performing bias, detection bias, and attrition bias when estimating health effects from evidence generated from non-randomised designs included in systematic-reviews. |  | Yes |
| Rosendaal | 2001 | [[43](#_ENREF_43)] | Cardiology | Case-control design is the best choice for the investigation of the etiology of diseases and the adverse events of drugs | Yes |  |
| Sharma | 2012 | [[44](#_ENREF_44)] | Social | Evidence-based medicine should integrate the patient's unique biology, and pathophysiology is evidence and should be regarded as such. Evidence is anything which provides justification for a belief, or which reduces uncertainty in the context of decision making. | Yes |  |
| Shrier | 2007 | [[45](#_ENREF_45)] | Various | NRS are more prone to bias because randomization removes the chance of confounding and the double-blind process minimizes biases caused by the placebo effect. Meta-analyses based on observational studies generally produce estimates of effect similar to those from meta-analyses based on randomized controlled trials. | Yes |  |
| Silverman | 2009 | [[46](#_ENREF_46)] | Pharma | Observational studies using large health care databases can complement findings from RCTs. Results from these designs can expand upon outcomes of RCTs because of the use of larger and more diverse patient populations with common comorbidities and longer follow-up periods. | Yes |  |
| Vandenbroucke | 1998 | [[47](#_ENREF_47)] | Various | All study designs in medicine should receive their proper place. Neither randomization nor blinding are guarantees against "willingness to please", which is a complex phenomenon that can result from the best and honest intentions. Outside sponsors reserve the contractual right to stop large trials without having to explain why. Medical history has shown repeatedly that erring is possible, despite the best of arguments. | Yes |  |
| Vandenbroucke | 2004 | [[48](#_ENREF_48)] | Various | Randomisation is needed for a fair comparison between treatments. NRS are essential for our knowledge about causes and pathogenesis (for example, genetic, environmental, or infectious causes of disease) as well as prognosis and diagnosis. | Yes |  |
| Vandenbroucke | 2008 | [[5](#_ENREF_5)] | Various | RCTs can solve problems of "confounding by indication" in situations where observational research can not. To measure the intended effects of treatment, we need "concealed randomisation". In contrast, adverse effects are "unintended effects" of treatment and they usually are not associated with the indications for treatment. RCTs are rarely used for research to detect or to establish causes of disease. | Yes |  |
| Vandenbroucke | 2009 | [[49](#_ENREF_49)] | HRT | The cardiovascular risk of HRT is real and slightly stronger in older women and the breast cancer risk or HRT is also real and is stronger in women closer to menopause. The results of NRCT and RCT fell in line mainly by analysing the data according to time. The reasons for initial discrepancies between RCTs and NRCT of risk estimates for coronary heart disease and breast cancer were rooted in the timing of HRT and not in differences in study design. Discrepancies between RCTs and NRCTs cannot be automatically attributed to randomization itself.   - Regarding coronary heart disease, RCTs observed the patients from the start of treatment and found an increase of coronary heart disease in the first years of use. NRCTs observed the patients in a later time period and did not find an increased risk. When cohort data from the NRCTs were reanalyzed according to time since start of therapy, the same pattern of an initial increase in risk followed by a decrease emerged in both study designs. - Regarding breast cancer, NRCTs observed patients that were close to menopause and found an increased risk for breast cancer. RCTs observed patients that were less close to menopause and did not find an increased risk. When the data from the RCTs were adjusted for the gap between menopause and treatment, an increase in risk was shown for both study designs. | Yes |  |
| Vandenbroucke | 2011 | [[50](#_ENREF_50)] | Various | Data from daily medical practice cannot be used to investigate the intended effects of treatments. Trials with concealed randomisation are needed to obtain the right answers. | Yes |  |
| Wilcken | 2001 | [[51](#_ENREF_51)] | Genetics | Rare diseases are not only rare but that they also have variable expression, long courses, and incompletely known late effects. RCTs are usually impossible because of inadequate power, and because there are preconceived notions of the effects of treatments already in use. The authors recommend the adoption of the best possible design for observational studies and the formation of a central registry of such trials. | Yes |  |
| Zlowodzki | 2006 | [[52](#_ENREF_52)] | Various | Several pitfalls in the design and conduct of clinical research include: lack of randomization, lack of concealment, lack of blinding, errors in hypothesis testing (type I and II errors), and unappropriate sample size considerations. |  | Yes |
| ***Systematic review*** |  |  |  |  | 6 | 2 |
| ***Non-systematic review*** |  |  |  |  | 30 | 4 |
| **Total** |  |  |  |  | 36 | 6 |
